# Height, autoimmune thyroid disease and thyroid cancer: a Mendelian randomisation study

**DOI:** 10.1089/thy.2023.0272

**Published:** 2023-09-29

**Authors:** Areti Papadopoulou, Bjørn O. Åsvold, Stephen Burgess, Aleksander Kuś, Marco Medici, Rosalie Sterenborg, Alexander Teumer, Panos Deloukas, Eirini Marouli

**Affiliations:** 1 William Harvey Research Institute, Faculty of Medicine and Dentistry, Queen Mary University of London, London, UK; 2 K.G. Jebsen Center for Genetic Epidemiology, Department of Public Health and Nursing, NTNU, Norwegian University of Science and Technology, Trondheim, Norway; 3 Department of Endocrinology, Clinic of Medicine, St. Olavs Hospital, Trondheim University Hospital, Trondheim, Norway; 4 MRC Biostatistics Unit, University of Cambridge, Cambridge, United Kingdom; 5 Cardiovascular Epidemiology Unit, Department of Public Health and Primary Care, University of Cambridge, Cambridge, United Kingdom; 6 Department of Internal Medicine and Endocrinology, Medical University of Warsaw, Warsaw, Poland; 7 Department of Epidemiology, Erasmus Medical Center, Rotterdam, The Netherlands; 8 Department of Internal Medicine, Academic Center for Thyroid Diseases, Rotterdam, The Netherlands; 9 Department of Internal Medicine, Radboud Institute for Health Sciences, Radboud University Medical Center, Nijmegen, The Netherlands; 10 Academic Center for Thyroid Diseases, Department of Internal Medicine, Erasmus Medical Center, Rotterdam, The Netherlands; 11 Department of Psychiatry and Psychotherapy, University Medicine Greifswald, Greifswald, Germany; 12 DZHK (German Center for Cardiovascular Research), Partner Site Greifswald, Greifswald, Germany; 13 Digital Environment Research Institute, Queen Mary University of London, London, UK

## Abstract

**Background:**

Increased height has been associated with increased risk of hypothyroidism or thyroid cancer in epidemiological studies. However, the potential causal association between height and hypothyroidism or thyroid cancer have not been thoroughly explored. Autoimmune thyroid disease (AITD) mainly presents as hypothyroidism, thus, we aim to evaluate the causal relationship between height as exposure and its association with AITD or thyroid cancer.

**Methods:**

Mendelian randomisation (MR) analyses were performed by using genetic instruments associated with height, which were selected from the largest genome-wide association meta-analysis for height in up to 5.4 million individuals. Summary-level data for AITD and thyroid cancer, including 30,234 and 3,001 cases respectively, were collected from the large available genome-wide association studies. Bidirectional MR was performed to test for reverse causal association between AITD and adult height.

**Results:**

MR analyses showed that increased genetically predicted height was associated with a 4% increased risk of AITD ([95% CI 1.02, 1.07], pvalue=1.99E-03), per 1 standard deviation (SD) increase in genetically predicted height. The bidirectional MR did not show any causal association between AITD and adult height. Additionally, increased genetically predicted height was associated with 15% increased risk of thyroid cancer ([95% CI 1.07, 1.23], pvalue=2.32E-04), per 1-SD increase in height. Sensitivity analysis confirmed the main results.

**Conclusions:**

This MR study showed that one standard deviation increased genetically predicted height was associated with increased risk of AITD and thyroid cancer. In contrast, there was no evidence of a causal association of genetically predicted AITD on height. These results could be further aiding the investigation of height-related pathways as a means for gaining new mechanistic insights into AITD and thyroid cancer.

## Introduction

The relationship between standing height and autoimmune thyroid disease (AITD) remains poorly understood and the underlying mechanisms behind these interactions remain unclear. The population-wide prevalence of AITD is around 5%, which is the highest prevalence of autoimmune diseases and mainly presents as hypothyroidism and the vast majority of hypothyroidism is caused by autoimmunity^1^. Recently, a phenome-wide association study (PheWAS) of height revealed that genetically predicted height was robustly associated with increased risk of hypothyroidism in European American individuals ^2^. Hypothyroidism is observed when a reduction of thyroid hormones (thyroxine (T4) and triiodothyronine (T3)) result in increased levels of thyroid-stimulating hormone (TSH). If hypothyroidism is left untreated, it can, among other issues, cause cardiac problems, with patients manifesting musculoskeletal, neurological and psychological symptoms, alongside infertility in females. In habitats with iodine sufficiency, Hashimoto’s autoimmune disease is the most common cause of hypothyroidism. The manifestation of hypothyroidism is contingent on genetic, environmental and non-modifiable factors ^3^. Disease prevalence varies depending on sex, with females being 10 times at higher risk than males ^3,4^. Hypothyroidism differentiates according to geographical region and type; clinical or subclinical. The National Health and Nutrition Examination Survey (NHANES III) in the US showed prevalence of clinical and subclinical hypothyroidism of 0.3% and 4.2%, respectively ^5^. Similar prevalence was reported in a European meta-analysis ^6^. Hypothyroidism is also a known risk factor of growth retardation ^7^.

A meta-analysis from 2015 showed that increased measured height is associated with increased risk of thyroid cancer ^8^. The aforementioned PheWAS in European Americans supported these findings; increased measured height was associated with increased risk of thyroid cancer, however the association was not robust to multiple testing correction ^2^. Thyroid cancer is the most common malignancy of the endocrine system and has several subtypes characterised by different severity and frequency. Multiple factors are responsible for the thyroid cancer manifestation, including genetic and environmental ^9^.

Recently, the largest meta-analysis of genome-wide association studies (GWAS) for standing height in over 5.4 million individuals reported 12,111 independently associated markers at genome-wide significance ^10^, which allowed us to undertake a well-powered Mendelian randomisation (MR) study to assess the causal role of adult height on the risk of AITD or thyroid cancer. Additionally, to examine for reverse causation, we explored the role of AITD on height.

## Materials and Methods

### Assumptions of MR and study design overview

MR employs instrumental variables to evaluate if there is causal association of an exposure with an outcome. To perform an MR analysis there are three basic assumptions: 1) the single-nucleotide polymorphisms (SNPs) employed as instrumental variables are strongly associated with the exposure; 2) the instrumental variables are not associated with any confounders and 3) the instrumental variables are associated with the outcome only via the exposure. The advantage of this method is that it mimics the randomisation of confounders in randomised clinical trials, since the genetic variants are assigned randomly at conception, avoiding the cost of clinical trials and employing statistical analysis using only summary level data. As a result, this method is more robust regarding confounding, either by environmental or lifestyle factors, and reverse causality ^11^.

The main analyses presented in this study evaluates the causal association of adult height, AITD and thyroid cancer. Additionally, we assessed whether genetic predisposition to AITD is causally associated with final height acquisition. Sensitivity analyses excluded instrumental variables associated with the outcome at pvalue<1E-02 to exclude any pleiotropic variants associated with both the exposure and the outcome.

### Instrumental variable selection

We employed 12,111 genetic variants associated with height from the latest cross-ancestry meta-analysis which account for 40% of phenotypic variance ^10^. For the bidirectional association of AITD and height, we assessed the effect of 94 genetic variants associated with AITD ^1^. The estimates for height associated variants were extracted from the 2022 Yengo et al GWAS ^10^. Proxies from the 2018 Yengo et al. ^12^ were used for the AITD SNPs, using 50,000 unrelated and randomly sampled European participants of UKB as the LD reference panel, r2=0.8 and 250 kb window. Lists with the genetic variants used as instruments are included in Supplementary Tables S1, S2, S3.

### Summary statistics and study population

The present MR study includes summary-level data from: 1) GIANT consortium and 23andMe, using up to 5.4 million individuals (76% had European ancestry) for height ^10^; 2) Iceland and UK Biobank for AITD (30,234 cases and 725,172 controls all European ancestry) ^1^, and 3) five cohorts of thyroid cancer (up to 3,001 cases and 287,550 controls all European ancestry) ^13^.

### Two-sample MR

We performed a two-sample MR employing genetic instruments from the GWAS summary data as described above. TwoSampleMR ^14^ and MRPRESSO ^15^ packages in R were implemented for the present analyses. An efficient and robust way to estimate the causal effect of the exposure on the outcome is by using a single genetic variant, in the ratio method. GWAS provides plenty of SNPs to be used as genetic instruments for MR analyses, thus the individual ratio estimates for each SNP can be incorporated into one more powerful estimate. This is called the inverse-variance weighted method (IVW), and it is formed as the weighted mean of the individual ratio estimates, where the weights equal the inverse-variances of the ratio estimates ^16^. This method is unbiased only when all genetic variants are valid IVs, an assumption which is difficult to hold given the plethora of the genetic instruments that are available from the GWAS. Therefore, we evaluate more methods as sensitivity analyses, even though their power to detect a causal effect is lower. One of them is the weighted median method (WM), which uses the weighted median of the individual ratio estimates, offering an unbiased causal estimate when at least half of the information comes from valid genetic variants ^17^. MR-Egger is a method that acknowledges that some of the IVs are invalid, and thus aims to predict the causal estimate by incorporating them. To this end, MR-Egger employs the IVW method with one key addition of an intercept term, which is tested for average directional pleiotropic effect. The assumption that MR-Egger needs to fulfil to provide an unbiased causal estimate is the Instrument Strength Independent of Direct Effect (InSIDE), which is a weaker assumption that substitutes the third assumption of MR. For the InSIDE assumption to be fulfilled, any pleiotropic effects of genetic variants need to be independent of genetic variant-exposure association ^18^. Lastly, the MR Pleiotropy RESidual Sum and Outlier (MR-PRESSO) is an outlier detection method, that provides a causal estimate after outlier removal. Briefly, after applying the IVW using all genetic variants, in turn, one by one the genetic variants are omitted from the analyses. Then, the residual sum of squares (RSS) is calculated, and if it is reduced substantially in comparison to the expected distribution, then the genetic variant is excluded. This process is repeated until none of the remaining genetic variants are considered as outliers. After outlier removal, IVW method is applied to estimate the causal effect ^15^.

### Bidirectional MR

Bidirectional MR was employed to test reverse causal direction. The aim of this analysis is to examine if genetic predisposition to AITD could affect adult height.

Institutional Review Board (IRB) approval (or waiver) statement: This study utilized summary statistics data, and no individual-level data were involved, ethical approval was not required.

## Results

### Height and AITD

**Figure 1:**
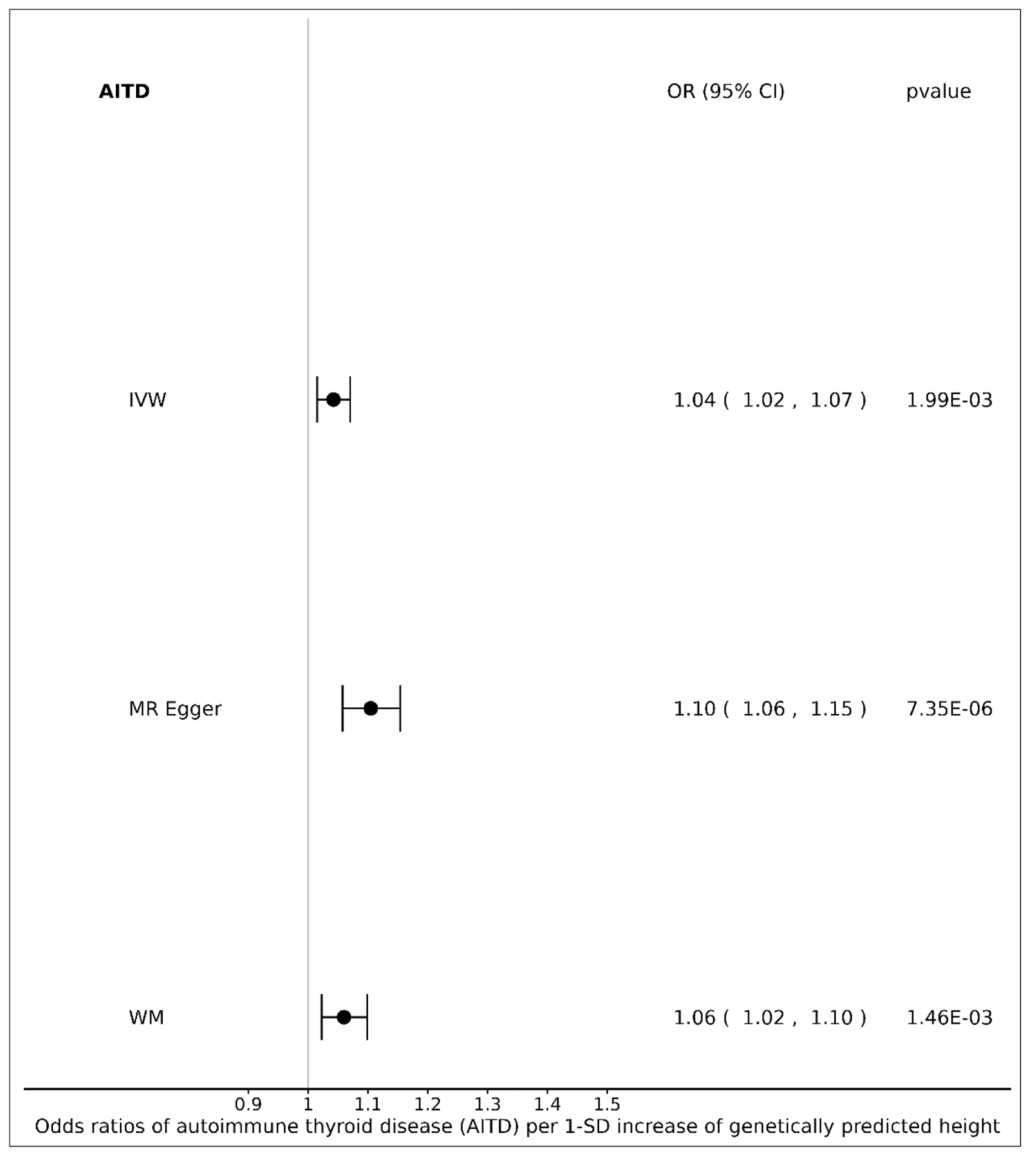
Forest plot: ORs for the effect of genetically predicted height on autoimmune thyroid disease (AITD) risk. CI, confidence interval; IVW, inverse variance weighted; MR, Mendelian randomization; OR, odds ratio; SD, standard deviation; WM, weighted median.

MR analyses suggest that one standard deviation (SD) increase in genetically predicted height was associated with an increased risk of AITD (OR=1.04 [95% CI 1.02, 1.07], pvalue=1.99E-03, IVW). This was supported by both WM and MR-Egger ([95% CI 1.02, 1.10], pvalue=1.46E-03 and [95% CI 1.06, 1.15], pvalue=7.35E-06 respectively) (Figure 1). Egger intercept was close to zero (*β*_0_ = -0.0008), but we cannot completely rule out the presence of directional horizontal pleiotropy (pvalue=1.03E-03, Supplementary Figure 1). Results from all MR methods are included in Supplementary Table S4.

We further performed sensitivity analysis excluding instrumental variables associated with the outcome at a pvalue<1E-02, to better honour the third MR assumption that genetic variables should influence the outcome only via the exposure. MR analyses with IVW suggested only a minor reduction in the effect in comparison to the main analysis with the signal remaining statistically significant; increased levels of genetically predicted height were associated with an increased risk of AITD (OR=1.03 [95% CI 1.014, 1.05], pvalue=7.84E-03). This was supported by WM and MR-Egger (Supplementary Figure 2, Supplementary Tables S4 and S5).

### Bidirectional MR

We employed bidirectional MR to test the possibility of reverse causation and examine whether genetically predicted AITD has an effect on adult height. MR analyses showed no evidence that genetic predisposition to AITD is associated with adult height (beta=-0.009 [95% CI -0.025, 0.006], pvalue=2.38E-01, IVW). The global test performed by MR-PRESSO, provided a statistically significant pvalue<5E-04, indicating the presence of horizontal pleiotropy. The outlier test identified some of the instrumental variables as outliers and excluded them. However the distortion test was not significant (pvalue=1.21E-01) (Supplementary Figure 3). Results from all MR methods are included in Supplementary Table S6.

We also performed bidirectional MR analyses after excluding variants associated with height at a pvalue<1E-02, which also showed that there was no evidence of causal association between AITD and adult height (beta=-0.003 [95% CI -0.010, 0.004], pvalue=4.00E-01, IVW). The global test performed by MR-PRESSO was not statistically significant (pvalue=6.60E-02), indicating that there was no evidence of horizontal pleiotropy (Supplementary Figure 4). Results from all MR methods are included in Supplementary Table S7.

### Height and thyroid cancer

**Figure 2:**
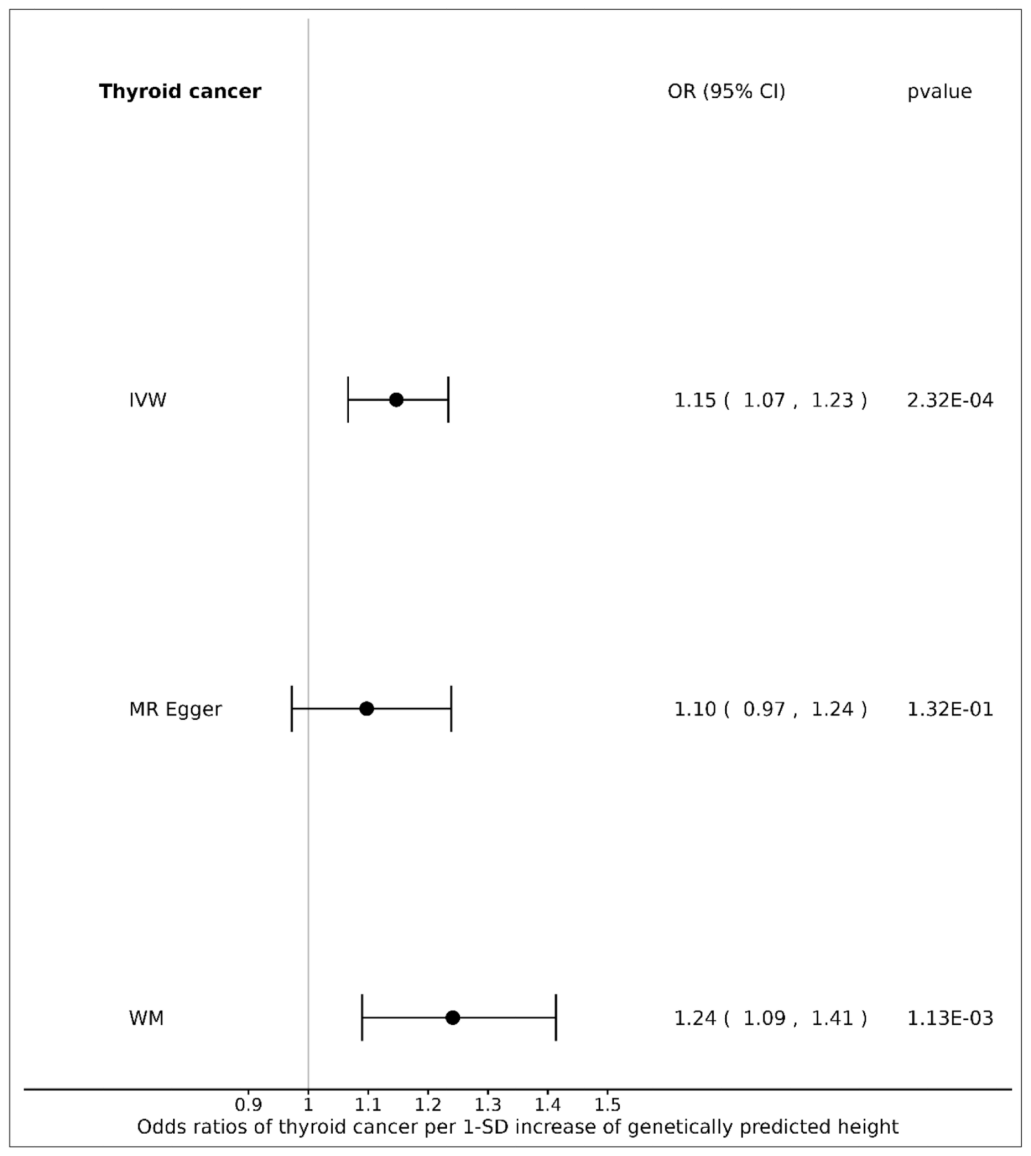
Forest plot: ORs for the effect of genetically predicted height on thyroid cancer risk. CI, confidence interval; IVW, inverse variance weighted; MR, Mendelian randomization; OR, odds ratio; SD, standard deviation; WM, weighted median.

MR analyses suggested that 1 SD increase in genetically predicted height was associated with an increased risk of thyroid cancer (OR=1.15 [95% CI 1.07, 1.23], pvalue=2.32E-04, IVW). WM detected a higher statistically significant risk ([95% CI 1.09, 1.41], pvalue=1.13E-03), compared to IVW, and had the same direction (Figure 2). MR-Egger confirmed the trend of increased risk of thyroid cancer; however the signal was not statistically significant. Furthermore, MR-Egger did not yield evidence of pleiotropy ([95% CI 0.97, 1.24], pvalue=3.72E-01) (Supplementary Figure 5). Results from all MR methods are included in Supplementary Table S8.

We further performed sensitivity analyses excluding instrumental variables associated with the outcome at pvalue<1E-02. MR analyses with IVW showed that the effect, although attenuated, remained significant; increased levels of genetically predicted height were associated with an increased risk of thyroid cancer (OR=1.12 [95% CI 1.05, 1.20], pvalue=1.28E-03, IVW). WM detected a greater statistically significant risk than IVW, having the same direction. MR-Egger estimated an increased risk of thyroid cancer as well, however not statistically significant ([95% CI 0.95, 1.19], pvalue=2.77E-01) (Supplementary Figure 6). The pvalue for the pleiotropy test for MR-Egger was not statistically significant, indicating no evidence of pleiotropy (pvalue=2.84E-01). Results from all MR methods are included in Supplementary Table S9.

## Discussion

In this study we used the most up to date list of genetic variants associated with adult height and large available summary statistics data for AITD and thyroid cancer to evaluate through MR the potential causal associations between adult height, AITD and thyroid cancer. We showed that increased genetically predicted height was significantly associated with both AITD and thyroid cancer, whereas there was no evidence for a reverse effect of AITD on height. These relationships remained statistically significant in the sensitivity analyses, where genetic variants associated with the outcome at a pvalue<1E-02 were excluded from the list of instrumental variables.

Our study indicates a causal association between adult height and AITD. The latest PheWAS of height revealed that measured height was robustly associated with increased risk of hypothyroidism in European American individuals (OR=1.04, pvalue=2.01E-06) ^2^. The same study though reported that increased genetically predicted height, using 3,290 SNPs from an earlier GIANT meta-analysis in Europeans ^12^, was associated with hypothyroidism, but this association was not statistically significant after multiple correction (OR=1.05, pvalue=3.76E-03) ^2^. Our findings are in agreement with the results of the PheWAS mentioned here for height. In the present study we use 4-fold more height genetic variants, which were derived from a cross-ancestry population ^10^, making it possible to present evidence for a causal relationship through the employed MR methods.

Children that present growth retardation should be examined regarding their thyroid hormone levels, as untreated hypothyroidism is a known risk factor of short stature. Therefore, exogenous replacement of thyroid hormones has been proposed to help children reach their predicted stature in adulthood, along with skeletal maturation, supporting the essential role of thyroid hormones in human development ^19^. Although most of the children that receive thyroid hormone replacement, reach their predicted height, there have been recorded cases who did not, underlying mechanisms that are connected to the duration and severity of hypothyroidism before diagnosis ^19,20^. However, an observational study in Italy showed that the age when the thyroid hormone treatment started did not have any role at the attainment of final height ^21^. In our analyses, the bidirectional MR showed no association of AITD with adult height; this result could be attributed to the fact that the GWAS used for AITD was performed in individuals that acquired AITD during adult life, since congenital hypothyroidism has been associated with growth retardation.

Adult height has also been associated with increased risk of several cancers, including thyroid cancer, however the association did not survive multiple testing correction in the latest height PheWAS ^2^. Additionally, a recent meta-analysis of observational studies reported that a 5-cm increase in adult height, increased the risk of thyroid cancer by 13% and 18% in males and females, respectively ^8^. The biological pathways underlying this effect are not fully elucidated. One possible explanation of the effect of height in thyroid carcinoma could be the role of insulin-like growth factor-1 (IGF-1). A recent systematic review of thyroid nodular disease endorsed the finding seen in many studies that the molecular pathway of IGF-1 is upregulated in thyroid carcinoma compared to benign nodes ^22^. This is also true for other type of cancers besides thyroid, including colorectal, breast and prostate ^23^. Increased IGF-1 levels could induce mutations in various cell lines, including thyroid cells, and contribute to the increment of cancer cases by stimulating cell proliferation, adhesion, and migration, and by inhibiting apoptosis. However, IGF-1 plays an important role in the development and regulation of postnatal growth, with taller people having increased levels of IGF-1 during childhood and adolescence, leading possibly to increased risk of thyroid cancer in adult life ^8,24^.

The analyses presented here have several advantages. Two-sample MR study design was used to eliminate unobserved confounding and reverse causality, however there was a sample overlap between height and AITD GWAS. The estimated bias and type I error in the MR analysis using height as exposure and AITD as outcome were 0.004 and 0.07, respectively ^25^. Thus, multiple MR methods were employed, and the concordance in findings provided additional confidence. Finally, we employed sensitivity analysis, by excluding SNPs strongly associated with the outcomes, and the associations, although attenuated, remained. Our study also had limitations. We employed proxies for the bidirectional analysis, since there was no full availability of public summary statistics data. However, the proxies we employed were looked up in a narrow window of 250 kb and were highly correlated with the original at r2=0.8. Also, we are employing summary statistics of adult height and AITD GWAS, and the most possible effects of hypothyroidism on height could be observed during childhood/adolescence. Future studies could further explore role of hypothyroidism and height in childhood or adolescence. Also, the height associated genetic variants used as instruments, were obtained after performing approximate conditional and joint (COJO) multiple-SNP analysis, as implemented in GCTA^10^. Additionally, our study was mainly performed in European ancestry individuals; GWAS for height included 76% Europeans, whereas AITD and thyroid cancer were performed in Europeans only. Thus, we cannot infer broader conclusions about ancestry specific effects. Through the exploration of the causal relationships presented here, our findings have the potential to support identification of individuals who might be at higher risk for AITD, or thyroid cancer based on their height or use height as a predictor, offering opportunities for more targeted screening, early detection, and preventive measures.

## Conclusions

We showed that increased genetically predicted height was associated with increased risk of AITD, and not vice versa. Furthermore, increased genetically predicted height was associated with increased risk of thyroid cancer. These results could be further aiding the investigation of height-related pathways as a means for gaining new mechanistic insights with potential clinical relevance into AITD and thyroid cancer.

## Supplementary Material

Supplementary material

Table S1

Table S2

Table S3

Table S4

Table S5

Table S6

Table S7

Table S8
