# Supplementary material for: Height, autoimmune thyroid disease and thyroid cancer: a Mendelian randomisation study
Source: Thyroid. Author manuscript; Available in PMC 2023 Dec 9. (PMC7615364; doi:10.1089/thy.2023.0272)
Supplement: Supplementary material [file EMS189053-supplement-Supplementary_material.docx]

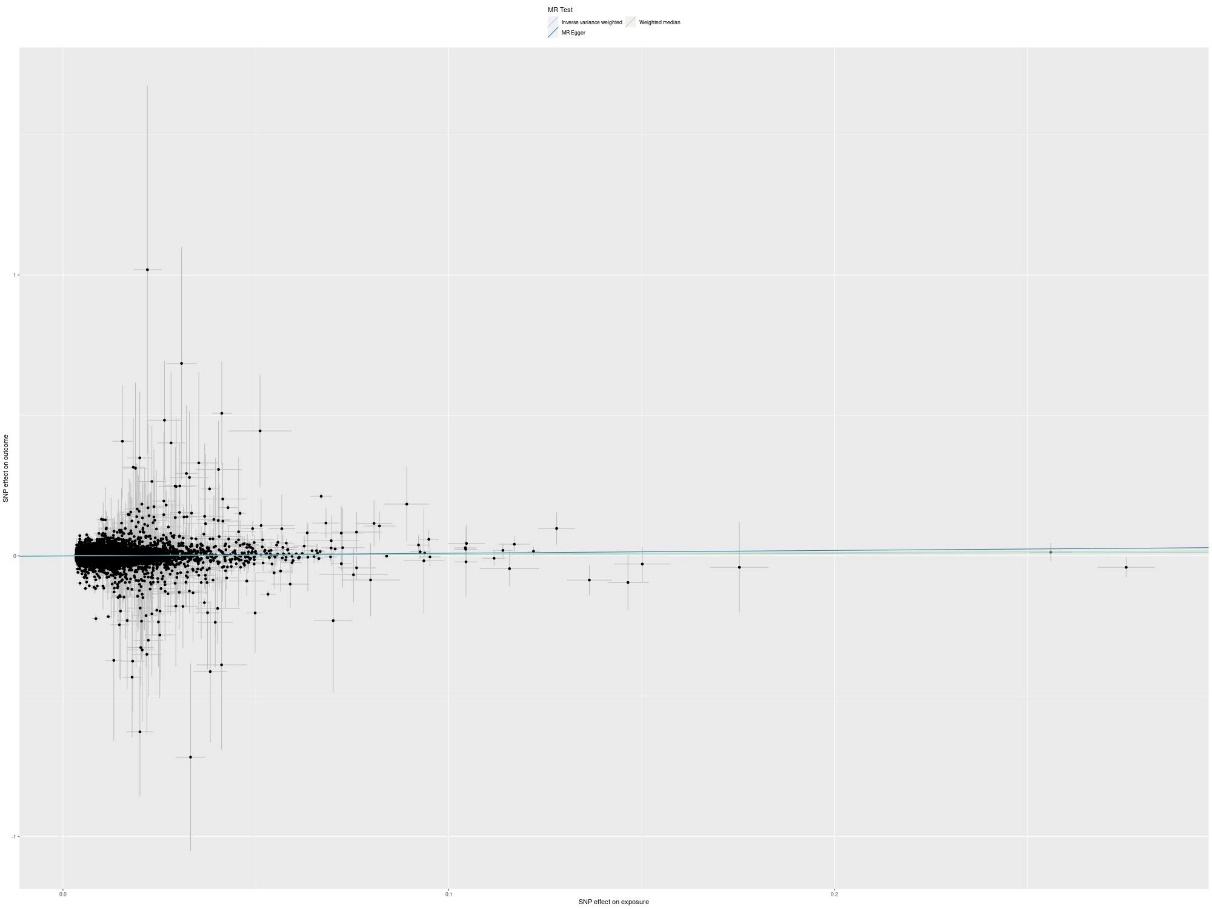


Supplementary Figure 1 Each point represents the SNP effects on height on the X-axis and autoimmune thyroid disease (AITD) on Y-axis. Coloured lines represent inverse-variance-weighted (grey), weighted median (green), and MR-Egger (blue) estimates of the association between a 1-SD increase in height and risk of AITD.


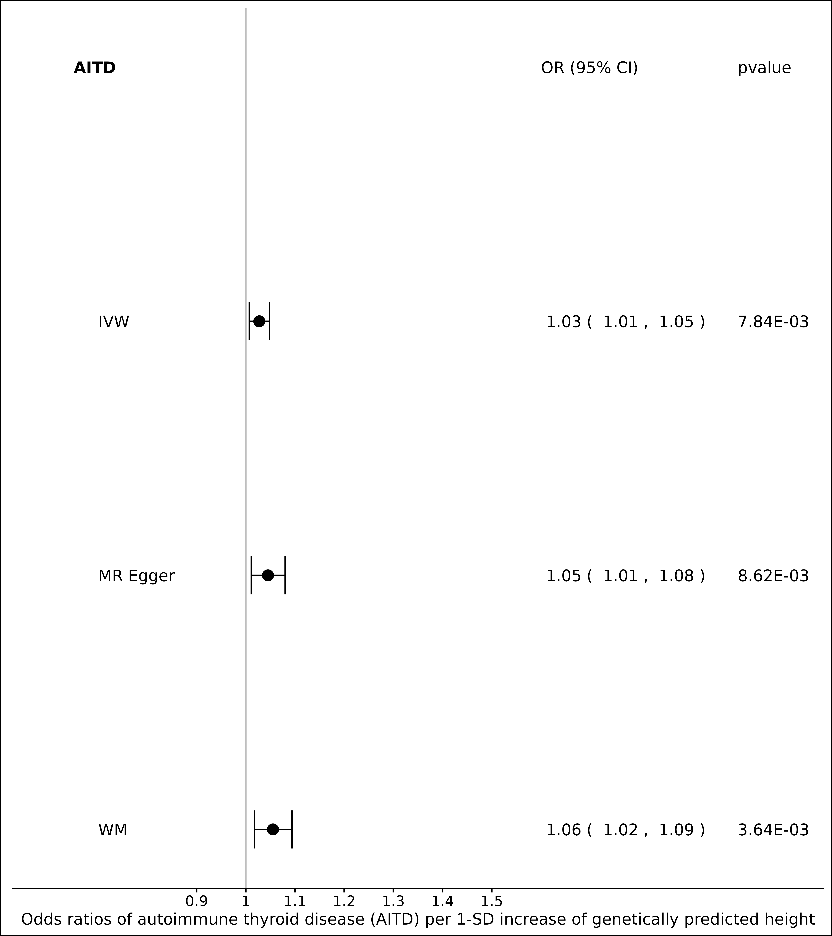


Supplementary Figure 2: Forest plot: ORs for the effect of genetically predicted height on autoimmune thyroid disease (AITD), after excluding IVs with pvalue<1E-02. CI, confidence interval; IVW, inverse variance weighted; MR, Mendelian randomization; OR, odds ratio; SD, standard deviation; WM, weighted median.


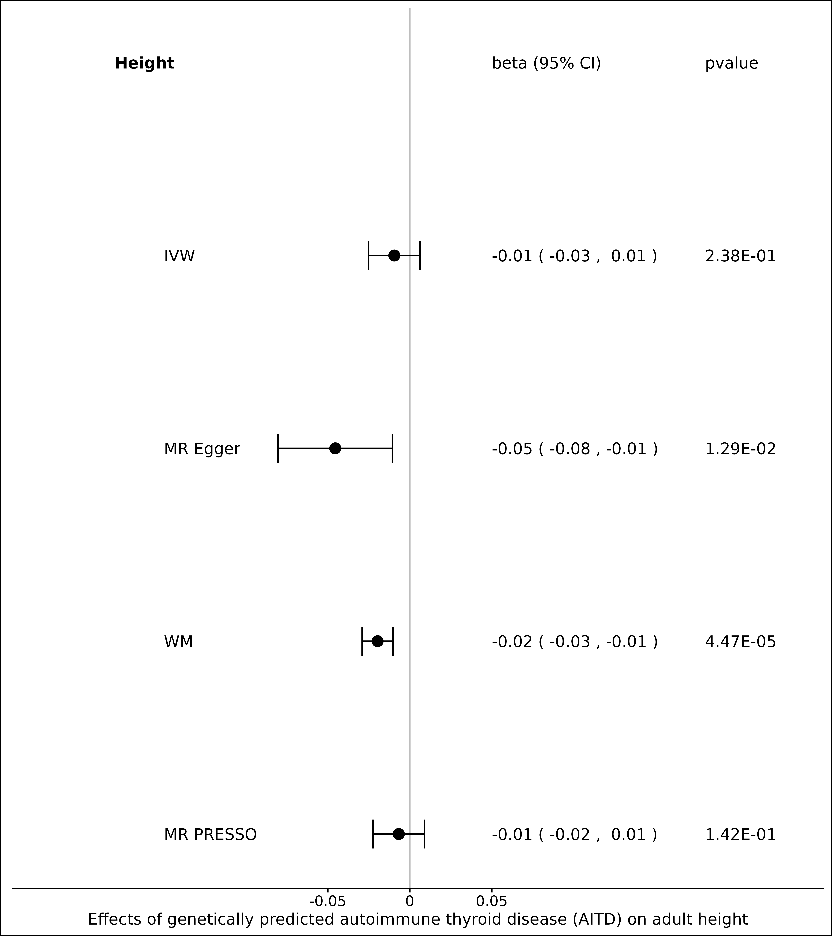


Supplementary Figure 3: Forest plot: Effects of genetically predicted autoimmune thyroid disease (AITD) on adult height. CI, confidence interval; IVW, inverse variance weighted; MR, Mendelian randomization; MR PRESSO: MR Pleiotropy RESidual Sum and Outlier; OR, odds ratio; WM, weighted median.


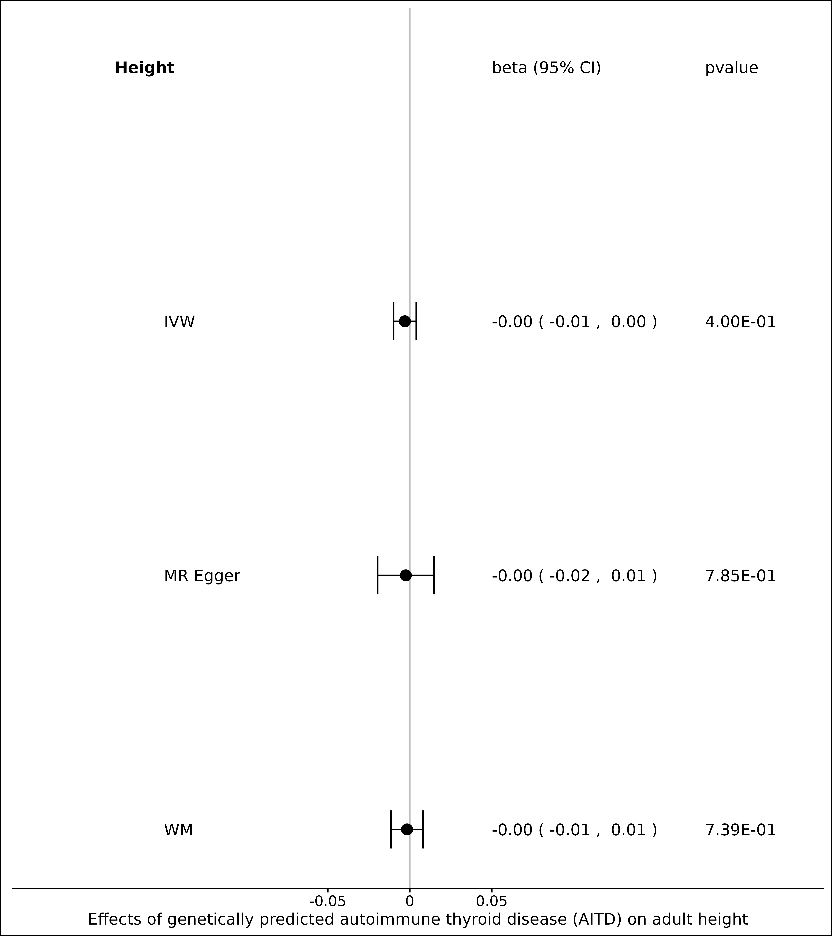


Supplementary Figure 4: Forest plot: Effects of genetically predicted autoimmune thyroid disease (AITD) on adult height, after excluding IVs with pvalue<1E-02. CI, confidence interval; IVW, inverse variance weighted; MR, Mendelian randomization; WM, weighted median.


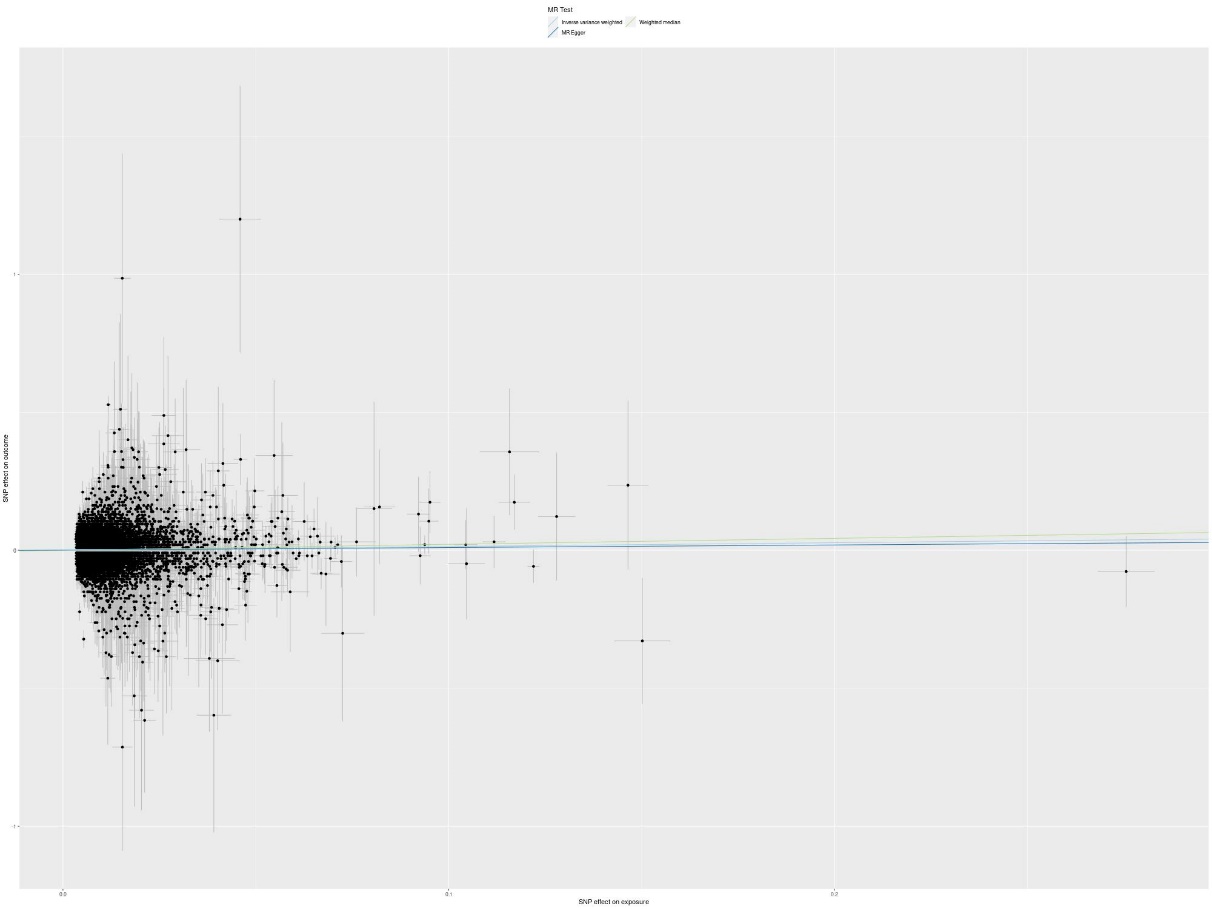


Supplementary Figure 5: Each point represents the SNP effects on height on the X-axis and thyroid cancer on Y-axis. Coloured lines represent inverse-variance-weighted (grey), weighted median (green), and MR-Egger (blue) estimates of the association between a 1-SD increase in height and risk of thyroid cancer.


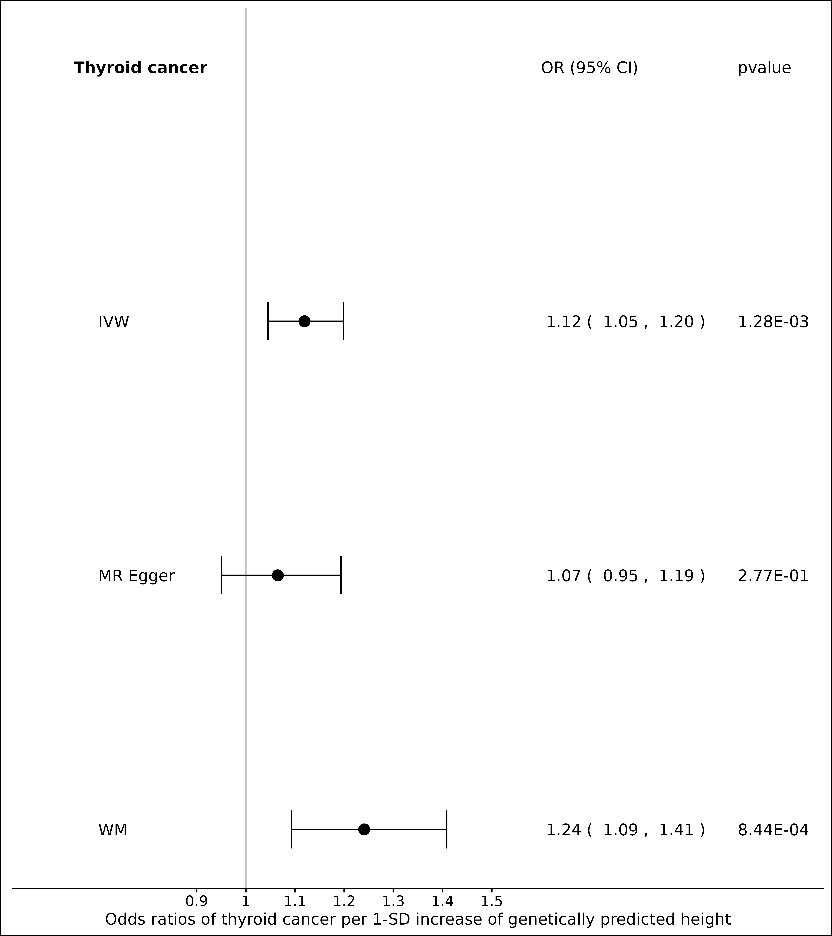


Supplementary Figure 6: Forest plot: ORs for the effect of genetically predicted height on thyroid cancer risk, after excluding IVs with pvalue<1E-02. CI, confidence interval; IVW, inverse variance weighted; MR, Mendelian randomization; OR, odds ratio; SD, standard deviation; WM, weighted median.
